# Exploring the relationship of platelet aggregation function with efficacy and safety outcomes following the administration of prasugrel and clopidogrel in patients with thrombotic stroke: a post hoc analysis of PRASTRO pooled studies

**DOI:** 10.1007/s11239-025-03093-3

**Published:** 2025-04-14

**Authors:** Kazumi Kimura, Masahiro Kamouchi, Yuji Matsumaru, Tetsuya Kimura, Rina Katsuro, Jun Hosokawa, Takanari Kitazono

**Affiliations:** 1 Department of Neurology, Graduate School of Medicine, Nippon Medical School, 1-1-5, Sendagi, Bunkyo-ku, Tokyo, 113-8603 Japan; 2 Department of Health Care Administration and Management, Graduate School of Medical Sciences, Kyushu University, Fukuoka, Japan; 3 Department of Neurosurgery, Faculty of Medicine, University of Tsukuba, Ibaraki, Japan; 4 Primary Medical Science Department, Daiichi Sankyo Co., Ltd, Tokyo, Japan; 5 Data Intelligence Department, Daiichi Sankyo Co., Ltd, Tokyo, Japan; 6 Department of Medicine and Clinical Science, Graduate School of Medical Sciences, Kyushu University, Fukuoka, Japan

**Keywords:** Clopidogrel, Large-artery atherosclerosis, Prasugrel, Small-vessel occlusion, Thrombotic stroke

## Abstract

The P2Y12 receptor inhibitor prasugrel was approved for thrombotic stroke in Japan following the phase 3 clinical trials PRASTRO-I, -II, and -III. However, correlations between elevated platelet reaction unit (PRU) and ischemic event risk remain unclear. This post hoc integrated analysis of PRASTRO-I, -II, and -III assessed the relationships of PRU with efficacy and safety outcomes, and risk factors for high PRU (HPR). Patients from PRASTRO-I, -II, and -III receiving prasugrel or clopidogrel and with PRU values at 4 and 24 weeks after treatment initiation were included. The primary endpoint was PRU at 4 weeks; secondary endpoints included cumulative incidence of ischemic and bleeding events from study drug initiation to 48 weeks. Exploratory univariate and multivariate analyses were conducted to identify HPR risk factors. Of 2688 patients analyzed, 2595 and 2434 had PRU values available at 4 and 24 weeks, respectively. Mean PRU was numerically lower with prasugrel than clopidogrel at 4 weeks (151.3 vs. 195.4) and 24 weeks (143.8 vs. 188.0). CYP2C19 polymorphisms affected PRU at 4 and 24 weeks with clopidogrel but not with prasugrel. PRU at 4 weeks did not predict ischemic and bleeding event incidence up to 48 weeks. The CYP2C19 poor metabolizer phenotype was the strongest HPR risk factor. PRU values at 4 and 24 weeks were numerically lower with prasugrel and unaffected by CYP2C19 genetic polymorphisms. Further research is needed to clarify the relationship of PRU with ischemic and bleeding events.

**Supplementary Information:**

The online version contains supplementary material available at 10.1007/s11239-025-03093-3.

## Clinical trial registration

jRCT2080221542 (PRASTRO-I), jRCT2080221856 (PRASTRO-II), jRCT2080224080 (PRASTRO-III)

## Graphical abstract

**Figure:**
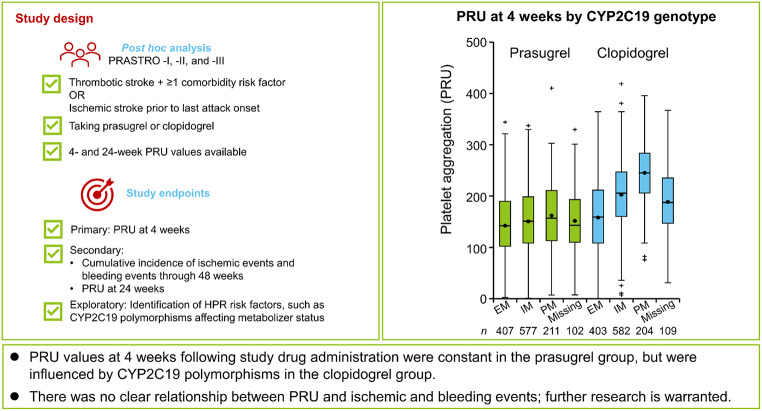
Study design, box-and-whisker plots showing median (IQR) PRU values by gene polymorphism at 4 weeks, and main study conclusions. •, arithmetic mean; +, values between 1.5*IQR and 3*IQR from UQ/LQ. EM, extensive metabolizer; HPR, high platelet reaction unit; IQR, interquartile range; IM, intermediate metabolizer; LQ, 25 percentile; PM, poor metabolizer; PRU, platelet reaction unit; UQ, 75 percentile

## Key points

• Four weeks after treatment initiation, thrombotic stroke patients taking prasugrel had lower PRU levels than those taking clopidogrel.

• CYP2C19 gene polymorphisms affected PRU levels in patients taking clopidogrel but not those taking prasugrel.

• PRU level did not predict incidence of ischemic and bleeding events.

• The ability of prasugrel to suppress PRU levels regardless of genetic polymorphisms suggests its utility in clinical practice.

## Introduction

Patients who have experienced a stroke have a high recurrence risk, and guidelines recommend antiplatelet therapy for the secondary prevention of non-cardioembolic stroke [1, 2]. Clopidogrel, a widely used antiplatelet agent, is a P2Y12 receptor inhibitor [3]. Metabolism from the prodrug to active metabolite relies heavily on CYP2C19; thus, its inhibitory effect on platelet aggregation is affected by CYP2C19 genetic polymorphisms [3]. Asian populations are especially affected by these polymorphisms, as a greater proportion of them are CYP2C19 poor metabolizers (PMs) and intermediate metabolizers (IMs) compared with White populations of European descent [4, 5]. Carriers of CYP2C19 loss-of-function alleles who receive clopidogrel are at higher risk of thrombotic events, including recurrent ischemic stroke, than non-carriers [6].

Prasugrel is an antiplatelet agent that, similar to clopidogrel, is a P2Y12 receptor inhibitor following conversion to its active metabolite [7]. Prasugrel was approved in Japan for the prevention of recurrence after thrombotic stroke (large-artery atherosclerosis or small-vessel occlusion) [7] based on three phase 3 clinical trials (PRASTRO-I, -II, and -III) [8–10]. Prasugrel metabolism is not affected by CYP2C19 gene polymorphisms, and therefore is expected to be a novel treatment option with consistent efficacy for thrombotic stroke regardless of CYP2C19 gene polymorphisms. A previous integrated analysis of the PRASTRO-I, -II, and -III trials showed that prasugrel tended to reduce the incidence of ischemic events compared with clopidogrel, particularly among CYP2C19 PMs [11].

Patients with ischemic heart disease commonly receive P2Y12 receptor inhibitors as standard treatment to prevent recurrent ischemic events. An elevated platelet reaction unit (PRU) level after percutaneous coronary intervention (PCI) has been identified as an increased risk factor for recurrent ischemic events in these patients [12, 13]. Relatedly, the ABCD-GENE (Age, Body Mass Index, Chronic Kidney Disease, Diabetes Mellitus, and Genotyping) score has been proposed as a risk factor for high PRU (HPR) [14].

In ischemic stroke, despite a few minor studies [15, 16], there are no definitive conclusions regarding the correlation between PRU and ischemic event risk. We hypothesized that PRU is associated with clinical outcomes in ischemic stroke. This exploratory post hoc analysis of the integrated analysis of PRASTRO-I, -II, and -III aimed to examine the relationships of PRU with efficacy and safety, and factors related to HPR in patients with thrombotic stroke.

## Methods

### Study design and patient population

This post hoc analysis included patients from PRASTRO-I (jRCT2080221542), -II (jRCT2080221856), and -III (jRCT2080224080) [8–11] who were compliant with the dosage and administration of prasugrel or clopidogrel outlined in the package insert [7]; had thrombotic stroke (classified as large-artery atherosclerosis or small-vessel occlusion according to the Trial of Org 10172 in Acute Stroke Treatment [TOAST] classification [17]) with at least one comorbidity risk factor (hypertension, dyslipidemia, diabetes, or chronic kidney disease) or a history of ischemic stroke prior to their last attack onset; and had PRU values measured at 4 and 24 weeks after treatment.

### Study endpoints

The primary endpoint was PRU at 4 weeks after study drug initiation; secondary endpoints included the cumulative incidence of ischemic events (composite of ischemic stroke, myocardial infarction, and death due to other vascular causes) and bleeding events (composite of life-threatening bleeding, major bleeding, and clinically relevant bleeding) from study drug initiation to 48 weeks, and PRU at 24 weeks. Platelet reactivity was determined using the VerifyNow P2Y12 assay (Accumetrics, San Diego, CA, USA) [18].

### Pharmacodynamic measurements

To assess platelet aggregation, venous blood (2.0 mL) was collected in the pretreatment period, 8 h after study drug administration, at weeks 4, 24, and 48, and in the final study week. Platelet aggregation results from patients who had received the study drug for at least 5 consecutive days were included in the analysis [19].

The frequencies of two CYP2C19 single-nucleotide polymorphisms (19154G > A and 17948G > A) were analyzed to understand their relationship with inhibition of platelet aggregation by the study drug. To accomplish this, leukocyte DNA was extracted from blood samples (2.0 mL) collected at the time of confirmed informed consent and before the end of week 8. An external gene analysis laboratory (LSI Medience Corporation, Tokyo, Japan) performed the genotyping.

### Statistical analysis

Given that this was an exploratory analysis, no power calculation was performed. Summary statistics and box-and-whisker diagrams were prepared to evaluate PRU values for each CYP2C19 polymorphism (extensive metabolizer [EM], IM, PM) and each time point (baseline, 4 weeks post-dose, 24 weeks post-dose, and the final time point of 48 weeks).

Receiver operating characteristic (ROC) analysis was performed to assess the association of PRU at 4 weeks with ischemic or bleeding events, and to calculate area under the curve (AUC) and PRU cutoff values based on the Youden Index (total population and by stroke type); analyses were also conducted for PRU at 24 weeks. If the lower limit of the 95% confidence interval (CI) of the AUC for ischemic events was > 0.5, the PRU cutoff value for ischemic events (PRU > 208 [12]) was used to divide patients into HPR and low PRU subgroups.

Univariate and multivariate analyses were conducted to identify risk factors for HPR status. Odds ratios (ORs) and 95% CIs were calculated using a logistic regression model. ABCD-GENE scores were determined according to Angiolillo et al. [14]. Variables included in the multivariate analysis were pre-defined from clinically important factors [11] and variables with *p* < 0.1 in the univariate analysis, and included the following: P2Y12-type (prasugrel or clopidogrel), age, body weight, sex, smoking status, duration from onset to the initiation of the study treatment, ischemic stroke type, medical history (ischemic stroke, cardiovascular disease, and chronic arteriosclerosis obliterans), concomitant medications (proton pump inhibitor, calcium blocker, and angiotensin receptor blocker), CYP2C19 phenotype, and risk factor (hypertension, diabetes mellitus, chronic kidney disease, and dyslipidemia).

Statistical analyses were performed using SAS version 9.4 or later (SAS institute Inc., Cary, NC, USA).

## Results

### Study population

**Fig. 1:**
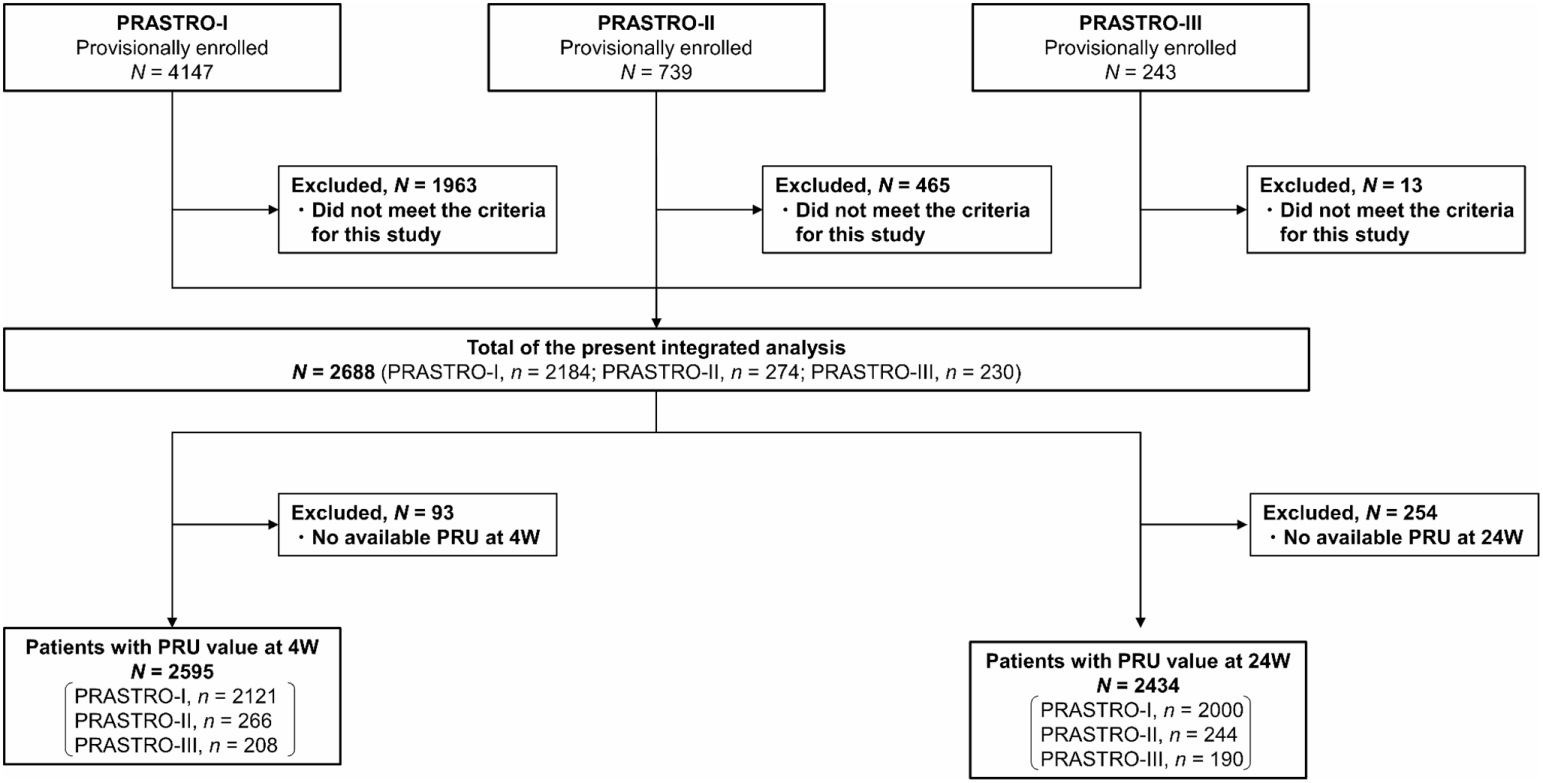
Patient disposition. PRU, platelet reaction unit; W, week

The integrated analysis included 2688 patients [11]; 2595 and 2434 had PRU values available at 4 and 24 weeks, respectively (Fig. 1). Baseline characteristics for patients with available PRU values at 4 weeks were similar between the groups (Table 1). The distribution of CYP2C19 polymorphism was 16.3%, 44.5%, and 31.4% in the prasugrel group and 15.7%, 44.8%, and 31.0% in the clopidogrel group for PMs, IMs, and EMs, respectively. Baseline characteristics for patients with PRU values at 24 weeks are shown in the table in Online Resource 1.

**Table 1:** Patient background (patients with a PRU value available at 4 weeks after starting treatment administration)

	Prasugrel (*N* = 1297)	Clopidogrel (*N* = 1298)
Mean ± SD, age, years	64.4 ± 9.4	64.7 ± 9.0
Male	1004 (77.4)	1008 (77.7)
Mean ± SD, bodyweight, kg	64.6 ± 10.8	64.6 ± 10.0
Mean ± SD, BMI, kg/m^2^	24.4 ± 3.3	24.3 ± 3.0
Time between onset of index stroke and trial treatment		
< 4 weeks	266 (20.5)	277 (21.3)
≥ 4 to < 12 weeks	598 (46.1)	624 (48.1)
≥ 12 weeks	432 (33.3)	397 (30.6)
Type of stroke		
Large-artery atherosclerosis	630 (48.6)	650 (50.1)
Small-vessel occlusion (lacunae)	667 (51.4)	648 (49.9)
Acute stroke of other determined etiology	0 (0.0)	0 (0.0)
Stroke of undetermined etiology	0 (0.0)	0 (0.0)
Modified Rankin Scale score		
0	307 (23.7)	329 (25.3)
1	686 (52.9)	701 (54.0)
2	209 (16.1)	202 (15.6)
3	68 (5.2)	38 (2.9)
4	27 (2.1)	28 (2.2)
5	0 (0.0)	0 (0.0)
History of atherosclerotic disease		
Ischemic stroke	193 (14.9)	183 (14.1)
Transient ischemic attack	87 (6.7)	81 (6.2)
Previously treated with clopidogrel	809 (62.4)	822 (63.3)
Comorbidities		
Hypertension	1108 (85.4)	1108 (85.4)
Dyslipidemia	899 (69.3)	921 (71.0)
Diabetes mellitus	451 (34.8)	475 (36.6)
Concomitant medication at baseline		
Statin	636 (49.0)	662 (51.0)
Insulin	51 (3.9)	47 (3.6)
Proton pump inhibitor	432 (33.3)	456 (35.1)
Calcium blocker	640 (49.3)	601 (46.3)
Angiotensin receptor blocker	654 (50.4)	650 (50.1)
Smoking status		
Never smoker	361 (27.8)	338 (26.0)
Former smoker	675 (52.0)	680 (52.4)
Current smoker	261 (20.1)	280 (21.6)
CYP2C19 phenotype		
Extensive metabolizer	407 (31.4)	403 (31.0)
Intermediate metabolizer	577 (44.5)	582 (44.8)
Poor metabolizer	211 (16.3)	204 (15.7)
Missing	102 (7.9)	109 (8.4)

Data are *n* (%) unless otherwise indicated. BMI, body mass index; PRU, platelet reaction unit; SD, standard deviation

### PRU according to CYP2C19 gene polymorphism

Mean ± standard deviation PRU at baseline, 4 weeks, and 24 weeks were 225.5 ± 81.1, 151.3 ± 64.4, and 143.8 ± 63.4, respectively, in the prasugrel group and 224.0 ± 83.2, 195.4 ± 74.2, and 188.0 ± 73.1 in the clopidogrel group (see the table in Online Resource 2).

**Fig. 2:**
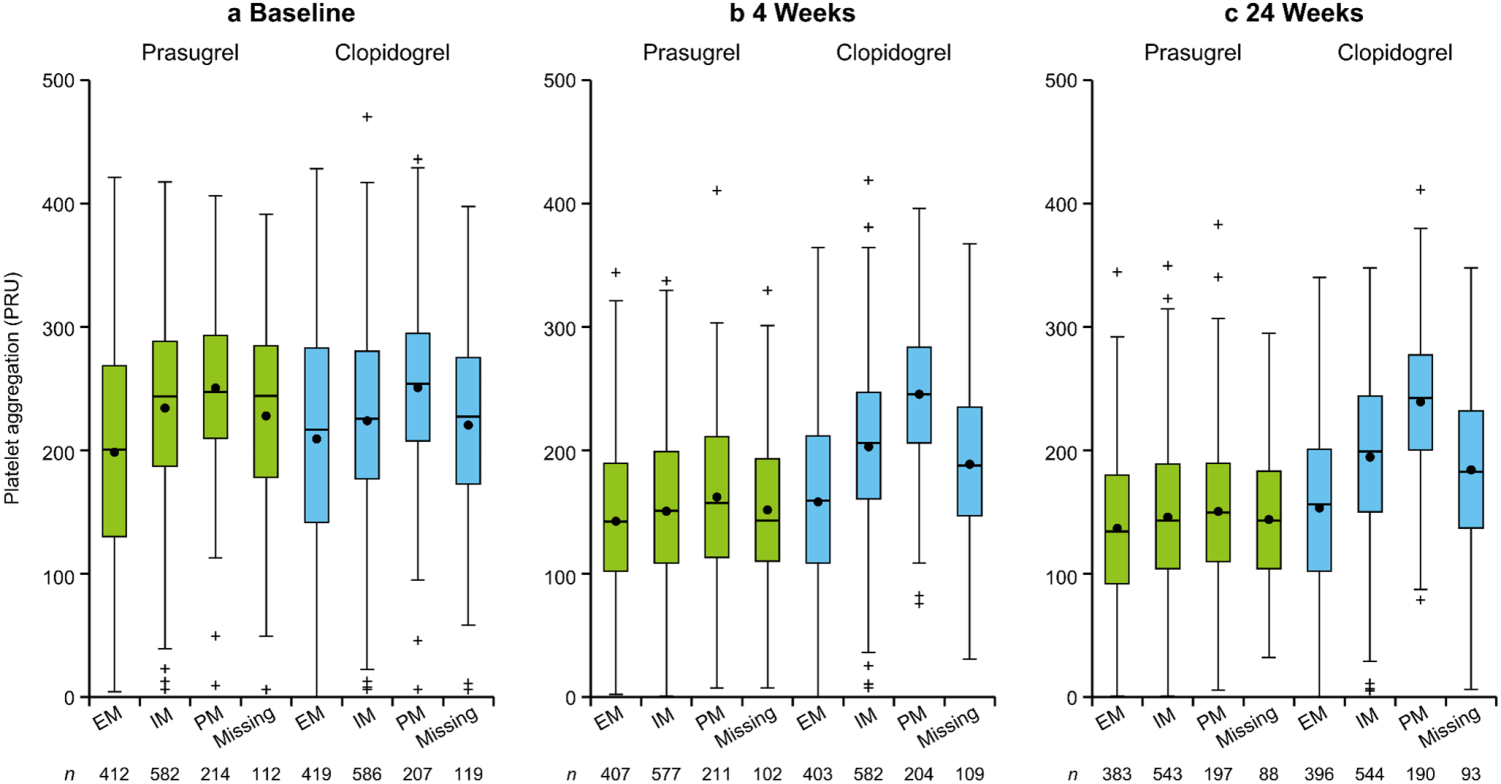
Box-and-whisker plots of PRU values by gene polymorphism at baseline (**a**), 4 weeks (**b**), and 24 weeks (**c**). •, arithmetic mean; +, values between 1.5*IQR and 3*IQR from UQ/LQ. EM, extensive metabolizer; IQR, interquartile range; IM, intermediate metabolizer; LQ, 25 percentile; PM, poor metabolizer; PRU, platelet reaction unit; UQ, 75 percentile

The primary endpoint, PRU at 4 weeks, was numerically lower with prasugrel than with clopidogrel. PRU at 4 weeks was similar for the EM, IM, and PM groups with prasugrel but was highest in the PM group, followed by the IM and EM groups with clopidogrel; PRU in the EM clopidogrel group was similar to that reported for all metabolizer groups with prasugrel (Fig. 2a, b). Similar trends were observed at 24 weeks (Fig. 2c).

### Predictive value of PRU for ischemic events and bleeding events based on ROC analysis

ROC curves of events from study drug initiation to 48 weeks are shown in the figure in Online Resource 3. Among patients with PRU values available at week 4 (*n* = 2595), 70 ischemic events and 93 bleeding events occurred, which decreased to 28 ischemic events and 69 bleeding events for PRU at week 24 (*n* = 2434) (see the table in Online Resource 4). AUC for PRU at week 4 was 0.529 (95% CI 0.462, 0.595) for ischemic events and 0.533 (95% CI 0.472, 0.593) for bleeding events; similar results were obtained when stratified by type of stroke and for PRU at 24 weeks (Table 2).

**Table 2:** AUC based on ROC analysis for events and PRUs at 4 and 24 weeks

	PRU value at 4 weeks	PRU value at 24 weeks
	AUC [95% CI]	AUC [95% CI]
**Ischemic events**		
All	0.529 [0.462, 0.595]	0.548 [0.435, 0.660]
Large-artery atherosclerosis	0.518 [0.441, 0.594]	0.550 [0.413, 0.688]
Small-vessel occlusion (lacunae)	0.539 [0.420, 0.657]	0.547 [0.374, 0.719]
**Bleeding events**		
All	0.533 [0.472, 0.593]	0.512 [0.443, 0.581]
Large-artery atherosclerosis	0.513 [0.415, 0.612]	0.544 [0.428, 0.660]
Small-vessel occlusion (lacunae)	0.542 [0.465, 0.619]	0.511 [0.425, 0.598]

AUC, area under the curve; CI, confidence interval; PRU, platelet reaction unit; ROC, receiver operating characteristic

### Analysis stratified by PRU cutoff values

Analysis stratified by PRU cutoff values was not performed because the lower 95% CI of the AUC for ischemic events was not > 0.5. Exploratory univariate and multivariate analyses were conducted using a cutoff value of PRU > 208 as reported previously (see the table in Online Resource 5) [12]. CYP2C19 phenotype PM was the strongest risk factor.

## Discussion

This exploratory post hoc analysis reports the findings of the first large-scale study of PRU in patients with thrombotic stroke (large-artery atherosclerosis or small-vessel occlusion) treated with prasugrel. PRU values at 4 weeks were numerically lower in the prasugrel than clopidogrel group. At 4 and 24 weeks, CYP2C19 gene polymorphism had no effect in the prasugrel group but had some effects in the clopidogrel group. In the ROC analysis of the association of 4-week PRU values with efficacy and safety events, the lower limit of the 95% CI for AUC was < 0.5, indicating that PRU at 4 weeks did not predict the incidence of ischemic and bleeding events from treatment initiation to 48 weeks. CYP2C19 phenotype PM was the strongest risk factor for HPR.

With prasugrel, PRU decreased from baseline and remained constant at 4 and 24 weeks regardless of CYP2C19 metabolizer type. With clopidogrel, PRU at 4 weeks differed, being highest in PM, followed by IM and EM; PRU values in the EM group were similar to those for all metabolizer groups with prasugrel. These findings are similar to those for patients undergoing PCI in the PRASFIT-ACS and PRASFIT-elective clinical studies [20, 21]. PM distribution in the present study was comparable to that in previous cardiology studies (18.6–28.1%) [20, 21], reiterating that CYP2C19 PM are common among Japanese patients with a history of stroke. Differential effects of CYP2C19 genetic polymorphisms on CYP2C19 inhibitor metabolism have been reported in several PCI studies [22–24], but similar reports in ischemic stroke patients are limited [25, 26]. The present study demonstrated that some CYP2C19 single-nucleotide polymorphisms affect platelet aggregation inhibition differently for prasugrel and clopidogrel in patients with ischemic stroke.

ROC analysis indicated that AUC for PRU values at 4 weeks (0.529 [0.462, 0.595]) did not exceed 0.5 of the lower 95% CI and did not predict recurrent stroke or other events up to 48 weeks. The same was true when classified by stroke type. Studies investigating associations between PRU values and recurrent stroke events are limited and have yielded mixed results [27]. The PRAISE study reported an association with PRU and recurrent acute ischemic stroke events among patients with large-artery atherosclerotic stroke but not with mid- to long-term ischemic stroke events [16]. Patients with PCI are relatively homogeneous, while patients with stroke tend to be more heterogeneous, with approximately half of patients having large-artery atherosclerosis and the other half having small-vessel occlusion. Thus, the relationship between PRU and stroke recurrence likely differs based on stroke etiology. Although PRU value did not predict prognosis (i.e., the occurrence of events) in the present study, we suggest that PRU values should continue to be monitored and managed considering the findings of other studies [16, 28]. It is possible that the effect size of these risk factors may evolve in relation to the duration following stroke onset. We have focused on platelet aggregation capacity as a risk factor for stroke recurrence that can be modified using antiplatelet agents. However, other risk factors (e.g., hypertension, dyslipidemia, diabetes, and chronic kidney disease) have also been reported.

Our findings related to the association between PRU and events differed from the PRAISE study, which examined the association between clinical outcomes and acute PRU in patients with ischemic stroke/transient ischemic attack and large-artery atherosclerosis. Using a PRU cutoff of 254, the authors found that acute PRU evaluation could predict recurrent ischemic stroke (particularly within 7 days) [16]. Differences between the two studies in time taken from event occurrence to drug administration may explain their disparate findings. The present findings are also inconsistent with those from a recent study on the association between high on-treatment platelet reactivity (HTPR) and clinical outcomes in patients with stroke treated with 75 mg clopidogrel or 100 mg aspirin [28]. HTPR was defined as PRU ≥ 208 for clopidogrel and ≥ 550 for aspirin. Although a significant association was observed between HTPR and the primary endpoint, the patients studied were those hospitalized with acute ischemic stroke within 14 days of onset, and the PRU value was measured on day 7 of treatment, which differed from the present study. The present study population included patients with acute (time between onset of index stroke and trial treatment < 4 weeks, approximately 20% of the study population) or chronic stroke (≥ 4 weeks, approximately 80%), and the time since stroke onset varied widely. Additionally, many patients dropped out after developing ischemic and bleeding events. For these reasons, the association between PRU and events may not have been fully evaluated. No relationship between PRU values and bleeding events was observed in the present study. However, given the study limitations, our results do not conclusively indicate that excessively lowering PRU is safe in clinical practice.

As an additional exploratory analysis, this study aimed to identify risk factors related to HPR using PRU > 208 as the criterion for HPR, as reported in the ADAPT-DES cardiac clinical study [12]. Multivariate analysis showed that the CYP2C19 phenotype PM and other risk factors (P2Y12 inhibitor [clopidogrel], body weight [> 50 kg], female, longer duration from onset to study treatment initiation, concomitant medications [proton pump inhibitor, calcium blocker, and angiotensin receptor blocker], and non-hypertension) were high-risk factors for HPR. Risk for HPR (PRU > 208) is reported as an ABCD-GENE score of ≥ 10 in cardiac studies [14]. Here, the univariate results (ABCD-GENE score ≥ 10 points, OR 1.99 [1.66, 2.40]) were similar to those previously reported [14]. However, the subscores showed that genotyping was the only significant factor; in contrast to the previous study, the other factors were not identified as risk factors [14]. As this was an exploratory analysis, further studies are needed to determine whether risk factors for HPR differ between patients with ischemic heart disease and those with thrombotic stroke. Genetic polymorphisms appear to have a common effect on HPR in stroke patients receiving antiplatelet medications, regardless of ischemic heart disease and thrombotic stroke. The results of this analysis do not provide a clinical implication for whether prasugrel or clopidogrel should be preferred. However, genetic polymorphisms are difficult to assess directly in clinical practice and it can take several days to obtain results. Given that the efficacy of prasugrel was less influenced by genetic polymorphisms than clopidogrel in this study, there may be advantages to administering prasugrel prior to obtaining genetic testing results. This should be clarified in future studies.

### Limitations

This study had several limitations. First, all limitations that applied to the original studies (PRASTRO-I, -II, and -III) also apply to the present study. Second, this was a post hoc analysis integrating data from three previous clinical trials. Third, many patients dropped out of the study after experiencing an ischemic or bleeding event. Thus, the 24-week PRU analysis population may have significant bias, limiting the ability to assess the relationship between PRU values and events. Fourth, the temporal relationship between PRU measurement and event onset was not considered. Fifth, data were not collected immediately after study drug administration or during the acute phase. Thus, it was not possible to evaluate the association between on-treatment PRU and coronary versus cerebral events. Sixth, the present analysis may have been underpowered: a power calculation was not performed given that this was an exploratory analysis. Seventh, although the PRU cutoff value was explored using ROC analysis, the effect of genetic polymorphisms was not taken into account. Therefore, a comprehensive analysis that ties together events, PRU levels, and CYP polymorphisms for prasugrel versus clopidogrel is needed. Eighth, this study only examined the utility of a single PRU value to predict outcomes. To fully understand their predictive potential, it may be necessary to evaluate the predictive ability of PRU values collected at different timepoints. Ninth, the use of different PRU cutoff values than those applied in this study may produce different results, and thus, the results of this study may not be generalizable. Finally, PRU values were based on when study drug administration was initiated, which resulted in a wide range of durations between stroke onset and study drug initiation. Changes in pathophysiology over time following stroke onset, which may have a significant effect on platelet aggregation function and event occurrence, were not considered in the study design and may have influenced the results. Therefore, the relationship between PRU and events should be further evaluated in larger prospective studies that account for time from stroke onset to PRU measurements/recurrent events.

## Conclusion

In this integrated analysis of PRASTRO-I, PRASTRO-II, and PRASTRO-III, PRU values at 4 weeks following study drug administration were constant in the prasugrel group, but were influenced by CYP2C19 genetic polymorphisms in the clopidogrel group. The analysis did not show a clear relationship of PRU with ischemic and bleeding events; further research is needed for clarification.

## Electronic supplementary material

Below is the link to the electronic supplementary material.

Supplementary Material 1

## Data Availability

Deidentified individual participant data and applicable supporting clinical study documents are available upon reasonable request at https://vivli.org/. In cases where clinical study data and supporting documents are provided pursuant to company policies and procedures, Daiichi Sankyo Co., Ltd. will continue to protect the privacy of clinical study participants. Details on data sharing criteria and the procedure for requesting access are available at https://vivli.org/ourmember/daiichi-sankyo/.
