# Supplementary material for: Exploring the relationship of platelet aggregation function with efficacy and safety outcomes following the administration of prasugrel and clopidogrel in patients with thrombotic stroke: a post hoc analysis of PRASTRO pooled studies
Source: J Thromb Thrombolysis. 2025 Apr 14;58(4):547–55. doi: 10.1007/s11239-025-03093-3 (PMC12043787; doi:10.1007/s11239-025-03093-3)
Supplement: Supplementary file 1 — Supplementary Material 1 [file 11239_2025_3093_MOESM1_ESM.pdf]

Journal of Thrombosis and Thrombolysis

Supplementary Material

Exploring the relationship of platelet aggregation function with efficacy and safety outcomes following the administration of prasugrel and clopidogrel in patients with thrombotic stroke: A post hoc analysis of PRASTRO pooled studies

Kazumi Kimura, Masahiro Kamouchi, Yuji Matsumaru, Tetsuya Kimura, Rina Katsuro, Jun Hosokawa, Takanari Kitazono

Corresponding Author: Kazumi Kimura, MD, PhD

Department of Neurology, Graduate School of Medicine, Nippon Medical School, 1-1-5, Sendagi, Bunkyo-ku, Tokyo 113-8603, Japan.

E-mail address: k-kimura@nms.ac.jp

Supplementary Table

Online Resource 1 Patient background (patients with a PRU value available at 24 weeks after starting treatment administration)

	Prasugrel (*N* = 1211)	Clopidogrel (*N* = 1223)
Mean ± SD, age, years	64.3 ± 9.3	64.5 ± 8.9
Male	934 (77.1)	947 (77.4)
Mean ± SD, bodyweight, kg	64.6 ± 10.8	64.6 ± 10.0
Mean ± SD, BMI, kg/m^2	24.4 ± 3.3	24.3 ± 3.0
Time between onset of index stroke and trial treatment		
<4 weeks	251 (20.7)	257 (21.0)
≥4 weeks to <12 weeks	554 (45.8)	587 (48.0)
≥12 weeks	405 (33.5)	379 (31.0)
Type of stroke		
Large-artery atherosclerosis	583 (48.1)	605 (49.5)
Small-vessel occlusion (lacunae)	628 (51.9)	618 (50.5)
Acute stroke of other determined etiology	0 (0.0)	0 (0.0)
Stroke of undetermined etiology	0 (0.0)	0 (0.0)
Modified Rankin Scale score		
0	296 (24.4)	312 (25.5)
1	633 (52.3)	655 (53.6)
2	199 (16.4)	194 (15.9)
3	60 (5.0)	36 (2.9)

4	23 (1.9)	26 (2.1)
5	0 (0.0)	0 (0.0)
History of atherosclerotic disease		
Ischemic stroke	173 (14.3)	171 (14.0)
Transient ischemic attack	83 (6.9)	78 (6.4)
Previously treated with clopidogrel	751 (62.0)	768 (62.8)
Comorbidities		
Hypertension	1035 (85.5)	1042 (85.2)
Dyslipidemia	838 (69.2)	869 (71.1)
Diabetes mellitus	427 (35.3)	440 (36.0)
Concomitant medication at baseline		
Statin	595 (49.1)	625 (51.1)
Insulin	48 (4.0)	43 (3.5)
Proton pump inhibitor	403 (33.3)	431 (35.2)
Calcium blocker	603 (49.8)	569 (46.5)
Angiotensin receptor blocker	604 (49.9)	615 (50.3)
Smoking status		
Never smoker	337 (27.8)	321 (26.2)
Former smoker	628 (51.9)	640 (52.3)
Current smoker	246 (20.3)	262 (21.4)
CYP2C19 phenotype		
Extensive metabolizer	383 (31.6)	396 (32.4)
Intermediate metabolizer	543 (44.8)	544 (44.5)
Poor metabolizer	197 (16.3)	190 (15.5)

Missing	88 (7.3)	93 (7.6)

Data are *n* (%) unless otherwise indicated. BMI, body mass index; PRU, platelet reaction unit; SD, standard deviation.

Supplementary Table

Online Resource 2 PRU according to CYP2C19 gene polymorphism

	Prasugrel					Clopidogrel				
	All	Phenotype: CYP2C19				All	Phenotype: CYP2C19			
		EM	IM	PM	Missing		EM	IM	PM	Missing
Baseline, *n*	1320	412	582	214	112	1331	419	586	207	119
Mean ± SD	225.5 ± 81.1	199.2 ± 90.1	234.5 ± 74.6	250.2 ± 65.2	228.9 ± 81.5	224.0 ± 83.2	210.0 ± 94.1	224.7 ± 77.7	251.5 ± 69.8	221.9 ± 78.3
Min, max	3, 420	4, 420	3, 417	7, 405	5, 391	0, 468	0, 427	1, 468	6, 433	6, 395
Median	237	200.5	243.5	247	243	229	216	225	255	228
(Q1, Q3)	(172, 284)	(129, 269)	(185, 287)	(209, 293)	(177.5, 285)	(168, 284)	(141, 283)	(176, 280)	(207, 295)	(172, 275)
PRU value at 4 weeks, *n*	1297	407	577	211	102	1298	403	582	204	109
Mean ± SD	151.3 ± 64.4	144.4 ± 61.9	151.8 ± 64.5	163.2 ± 67.3	151.9 ± 65.3	195.4 ± 74.2	159.1 ± 73.1	203.6 ± 68.6	245.8 ± 61.0	191.2 ± 62.2
Min, max	0, 408	3, 342	0, 336	7, 408	8, 327	1, 417	3, 364	1, 417	73, 396	31, 367
Median	150	143	153	158	144	201	161	207	247.5	188
(Q1, Q3)	(107, 197)	(101, 191)	(108, 199)	(113, 211)	(109, 193)	(148, 244)	(110, 212)	(160, 247)	(205, 283.5)	(146, 236)
PRU value at	1211	383	543	197	88	1223	396	544	190	93

24 weeks, *n*										
Mean ± SD	143.8 ± 63.4	137.1 ± 63.9	145.3 ± 63.3	152.2 ± 64.5	144.7 ± 56.7	188.0 ± 73.1	154.3 ± 70.8	195.1 ± 67.3	240.1 ± 59.1	183.9 ± 68.8
Min, max	2, 381	2, 343	2, 348	7, 381	32, 295	1, 410	1, 340	3, 348	78, 410	7, 348
Median	143	137	144	151	144	192	157	200	243	184
(Q1, Q3)	(100, 187)	(91, 181)	(104, 189)	(109, 191)	(103.5, 183.5)	(139, 243)	(102.5, 201.5)	(150.5, 244)	(200, 278)	(136, 232)

EM, extensive metabolizer; IM, intermediate metabolizer; PM, poor metabolizer; PRU, platelet reaction unit; Q, quartile; SD, standard deviation.

Supplementary Figure

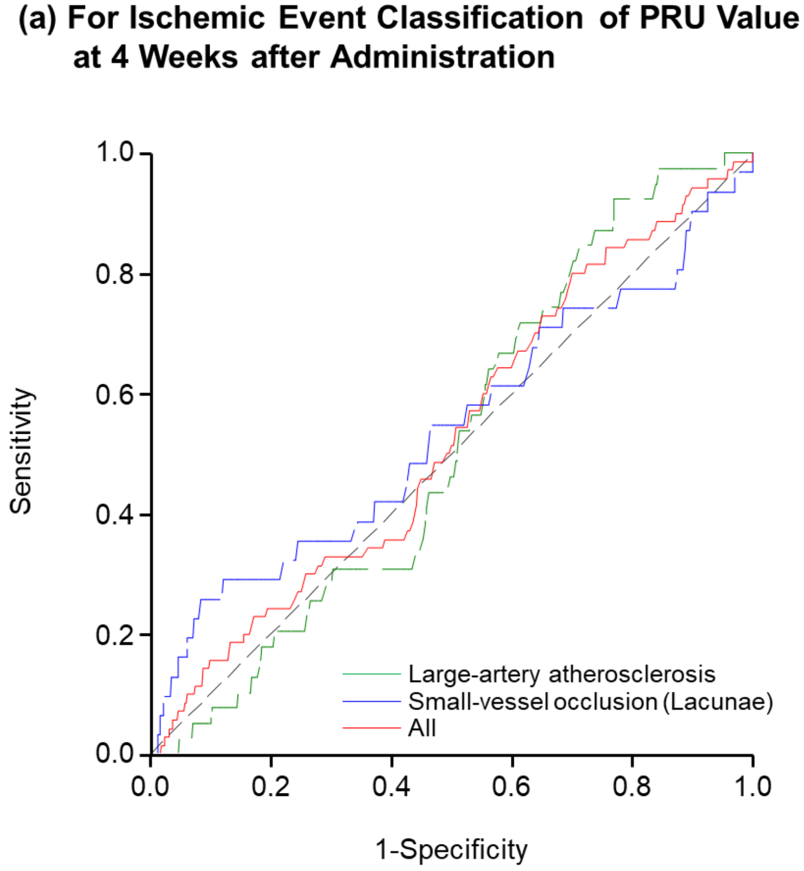

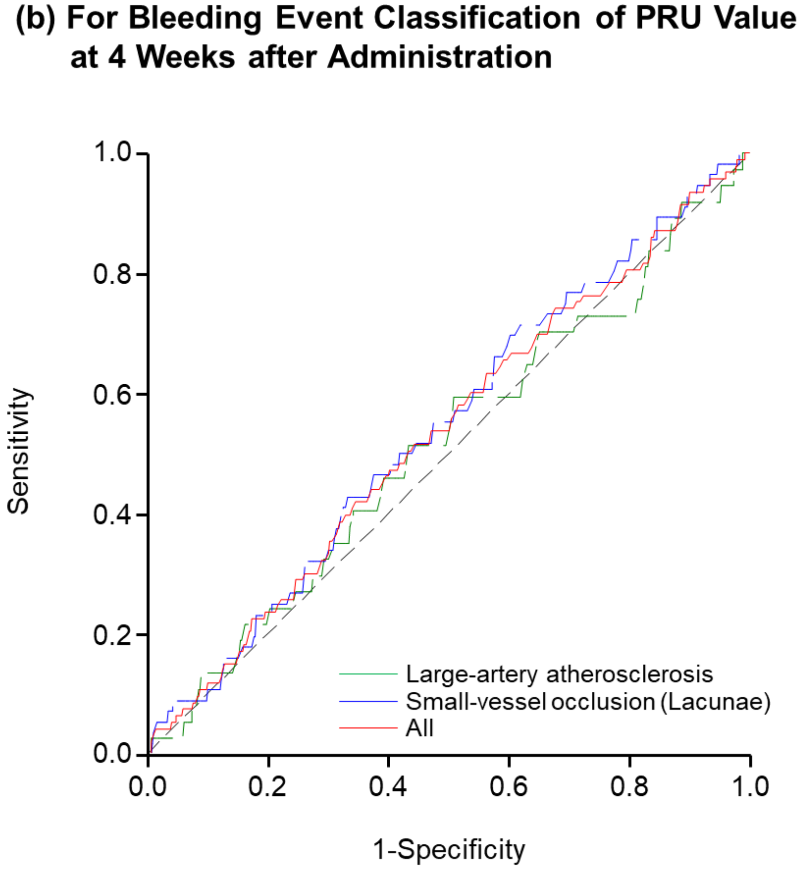

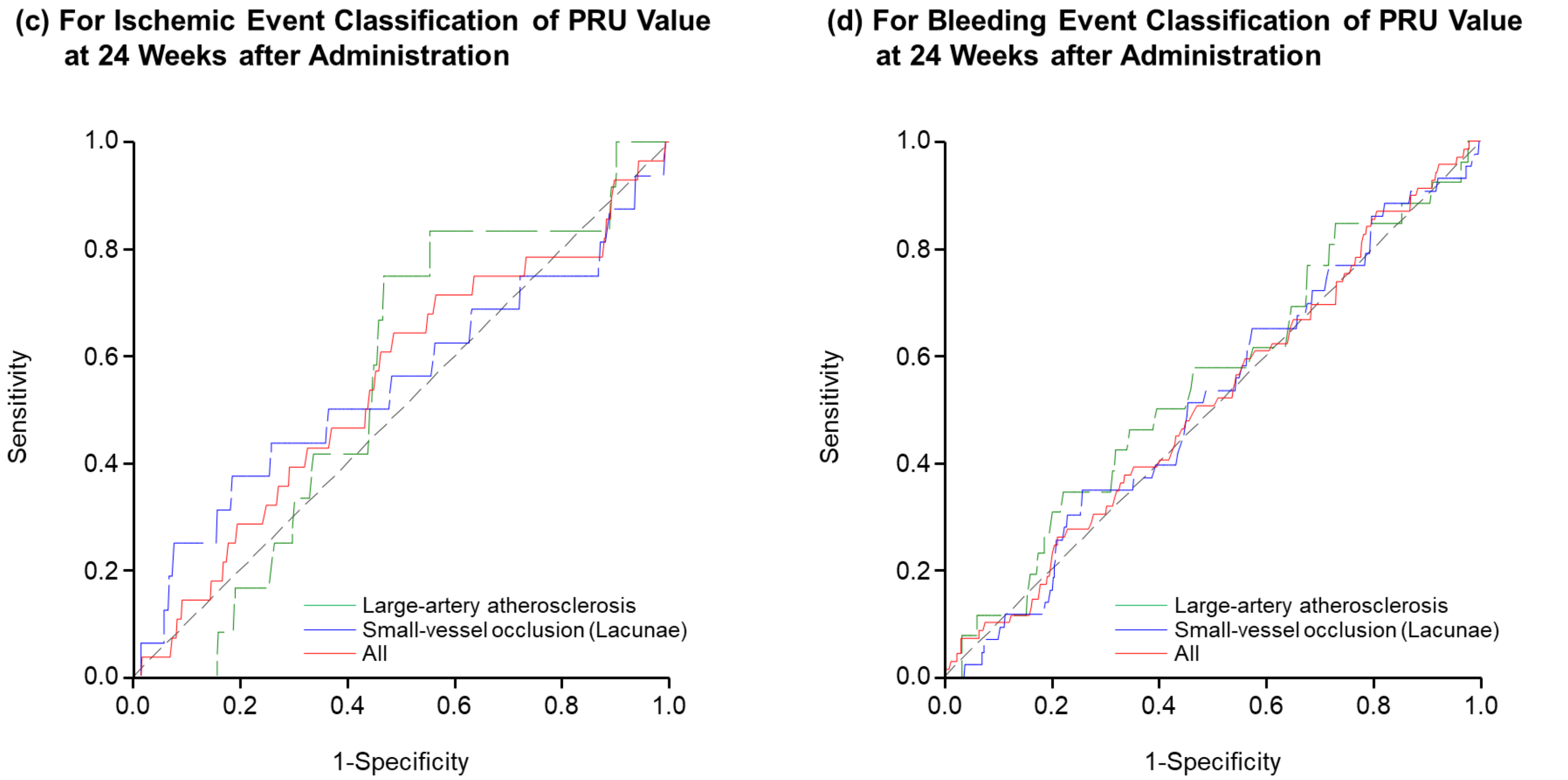

Online Resource 3 ROC analysis for ischemic event classification of PRU values at 4 weeks (**a**), bleeding event classification of PRU values at 4 weeks (**b**), ischemic event classification of PRU values at 24 weeks (**c**), and bleeding event classification of PRU values at 24 weeks (**d**). PRU, platelet reaction unit; ROC, receiver operating characteristic

Supplementary Table

Online Resource 4 Events targeted in ROC analysis

	Total of the integrated analysis		Patients with PRU value at 4 weeks		Patients with PRU value at 24 weeks	
	N	*Event n* (%) [95% CI]	*N*	*Event n* (%) [95% CI]	*N*	*Event n* (%) [95% CI]
**Ischemic events**						
All	2688	80 (3.0) [2.4, 3.7]	2595	70 (2.7) [2.1, 3.4]	2434	28 (1.2) [0.8, 1.7]
Large-artery atherosclerosis	1326	46 (3.5) [2.6, 4.6]	1280	39 (3.0) [2.2, 4.1]	1188	12 (1.0) [0.5, 1.8]
Small-vessel occlusion (lacunae)	1362	34 (2.5) [1.7, 3.5]	1315	31 (2.4) [1.6, 3.3]	1246	16 (1.3) [0.7, 2.1]
**Bleeding events**						
All	2688	98 (3.6) [3.0, 4.4]	2595	93 (3.6) [2.9, 4.4]	2434	69 (2.8) [2.2, 3.6]
Large-artery atherosclerosis	1326	40 (3.0) [2.2, 4.1]	1280	37 (2.9) [2.0, 4.0]	1188	26 (2.2) [1.4, 3.2]

Small-vessel occlusion (lacunae)	1362	58 (4.3) [3.2, 5.5]	1315	56 (4.3) [3.2, 5.5]	1246	43 (3.5) [2.5, 4.6]

CI, confidence interval; PRU, platelet reaction unit; ROC, receiver operating characteristic.

Supplementary Table

Online Resource 5 Exploratory analysis of risk factors for HPR at PRU 4 weeks (PRU cutoff >208)

	Univariate		Multivariate	
	OR [95% CI]	*p* value	OR [95% CI]	*p* value
P2Y12 inhibitor				
Prasugrel (vs Clopidogrel)	0.28 [0.24, 0.34]	<0.001	0.25 [0.21, 0.30]	<0.001
Age				
≥75 years (vs <75 years)	1.17 [0.90, 1.53]	0.234	1.25 [0.92, 1.70]	0.153
Body weight				
≤50 kg (vs >50 kg)	0.72 [0.48, 1.07]	0.105	0.49 [0.31, 0.78]	0.003
Sex				
Male (vs Female)	0.69 [0.57, 0.83]	<0.001	0.61 [0.49, 0.77]	<0.001
Smoking status				
Current (vs Never or Former)	0.85 [0.69, 1.05]	0.135	0.91 [0.73, 1.15]	0.449
Most recent ischemic stroke				
Revascularization procedure for the most recent ischemic stroke, Yes (vs No)	0.78 [0.57, 1.07]	0.130	-	-
Duration from onset to the initiation of the study treatment				
≥4 weeks, <12 weeks (vs <4 weeks)	1.65 [1.31, 2.08]	0.014	1.73 [1.34, 2.24]	0.013
≥12 weeks (vs <4 weeks)	1.77 [1.39, 2.26]	<0.001	1.86 [1.42, 2.45]	0.001

Classification of subtypes of ischemic stroke				
Large-artery atherosclerosis (vs small-vessel occlusion [lacunae])	1.20 [1.02, 1.42]	0.028	1.18 [0.98, 1.42]	0.076
Modified Rankin Scale score				
Grade 4 (vs Grade 0 to Grade 3)	0.96 [0.54, 1.70]	0.878	-	-
Grade 5 (vs Grade 0 to Grade 3)	-	-	-	-
Medical history				
Atherosclerotic disease				
Yes (vs No)	1.00 [0.82, 1.23]	0.965	-	-
Ischemic stroke				
Yes (vs No)	1.05 [0.83, 1.32]	0.703	1.08 [0.84, 1.40]	0.551
TIA				
Yes (vs No)	0.96 [0.68, 1.34]	0.794	-	-
Ischemic stroke / TIA				
Yes (vs No)	1.00 [0.81, 1.23]	0.993	-	-
Cardiovascular disease				
Yes (vs No)	0.97 [0.51, 1.82]	0.913	0.82 [0.41, 1.65]	0.582
Chronic arteriosclerosis obliterans				
Yes (vs No)	1.07 [0.53, 2.15]	0.848	1.03 [0.49, 2.17]	0.944
eGFR before drug administration				
<60 mL/min/1.73 m^2 (vs ≥60 mL/min/1.73 m^2)	1.10 [0.89, 1.34]	0.379	-	-
Creatinine clearance before drug administration				

Moderate (vs Normal or Mild)	0.92 [0.63, 1.37]	0.148	-	-
Severe (vs Normal or Mild)	2.14 [0.80, 5.73]	0.116	-	-
Prior treatment of clopidogrel				
Yes (vs No)	1.11 [0.93, 1.31]	0.249	-	-
Concomitant drug at the start of study drug administration				
Proton pump inhibitor				
Yes (vs No)	1.12 [0.95, 1.34]	0.183	1.22 [1.01, 1.48]	0.041
Calcium blocker				
Yes (vs No)	1.44 [1.22, 1.70]	<0.001	1.58 [1.29, 1.93]	<0.001
Statin				
Yes (vs No)	0.98 [0.83, 1.16]	0.823	-	-
Angiotensin receptor blocker				
Yes (vs No)	1.31 [1.11, 1.54]	0.002	1.28 [1.05, 1.57]	0.017
Insulin preparation				
Yes (vs No)	1.25 [0.82, 1.90]	0.293	-	-
Phenotype: CYP2C19				
IM (vs EM)	2.02 [1.64, 2.49]	0.119	2.27 [1.81, 2.83]	0.054
PM (vs EM)	3.69 [2.85, 4.77]	<0.001	4.41 [3.34, 5.82]	<0.001
Missing (vs EM)	1.46 [1.03, 2.06]	0.071	1.49 [1.03, 2.17]	0.036
IM + PM (vs EM)	2.38 [1.96, 2.91]	<0.001	-	-
Missing (vs EM)	1.46 [1.03, 2.06]	0.728	-	-
Risk factor				
Hypertension				
Yes (vs No)	1.06 [0.83, 1.34]	0.654	0.66 [0.49, 0.90]	0.009

Diabetes mellitus				
Yes (vs No)	1.11 [0.94, 1.32]	0.222	1.10 [0.91, 1.33]	0.336
Chronic kidney disease				
Yes (vs No)	1.14 [0.95, 1.36]	0.168	1.06 [0.87, 1.30]	0.557
Dyslipidemia				
Yes (vs No)	1.16 [0.81, 1.66]	0.414	1.14 [0.77, 1.70]	0.500
ABCD-GENE score				
≥10 (vs <10)	1.99 [1.66, 2.40]	<0.001	-	-

Variables included in the multivariate analysis were pre-defined from clinically important factors [1] and variables with $p < 0.1$ in the univariate analysis and included the following: P2Y12-type (prasugrel or clopidogrel), age, body weight, sex, smoking status, duration from onset to the initiation of the study treatment, ischemic stroke type, medical history (ischemic stroke, cardiovascular disease, and chronic arteriosclerosis obliterans), concomitant medications (proton pump inhibitor, calcium blocker, and angiotensin receptor blocker), CYP2C19 phenotype, and risk factor (hypertension, diabetes mellitus, chronic kidney disease, and dyslipidemia). *P* values were calculated using the chi-squared test. CI, confidence interval; eGFR, estimated glomerular filtration rate; EM, extensive metabolizer; HPR, high PRU; IM, intermediate metabolizer; OR, odds ratio; PM, poor metabolizer; PRU, platelet reaction unit; TIA, transient ischemic attack.

Reference

1. Kitazono T, Kamouchi M, Matsumaru Y, Shirai T, Takita A, Kuroda T, Kimura K. (2023)

Comparison of prasugrel and clopidogrel in thrombotic stroke patients with risk factors for

ischemic stroke recurrence: an integrated analysis of PRASTRO-I, PRASTRO-II, and PRASTRO-III.

Cerebrovasc Dis. 52:720–729. https://doi.org/10.1159/000529149
